# Identification of metabolite biomarkers in serum of rats exposed to chlorpyrifos and cadmium

**DOI:** 10.1038/s41598-020-61982-4

**Published:** 2020-03-19

**Authors:** Ming-Yuan Xu, Pan Wang, Ying-Jian Sun, Lin Yang, Yi-Jun Wu

**Affiliations:** 1 Laboratory of Molecular Toxicology, State Key Laboratory of Integrated Management of Pest Insects and Rodents, Institute of Zoology, Chinese Academy of Sciences, Beijing, 100101 P.R. China; 2 Department of Veterinary Medicine and Animal Science, Beijing University of Agriculture, Beijing, 102206 P.R. China

**Keywords:** Biochemistry, Systems biology

## Abstract

Chlorpyrifos (CPF) and cadmium (Cd) are widespread environmental pollutants, which are often present in drinking water and foods. However, the combined effects of CPF and Cd were not entirely clear at present. There was also no biomarker available to diagnose the poisoning of the two chemicals at low dose for long-term exposures. In this study, we investigated the change of serum metabolites of rats with subchronic exposure to CPF, Cd, and CPF plus Cd using gas chromatography-mass spectrometer-based metabolomics approach. We performed a stepwise optimization algorithm based on receiver operating characteristic to identify serum metabolite biomarkers for toxic diagnosis of the chemicals at different doses after 90-day exposure. We found that aminomalonic acid was the biomarker for the toxicity of Cd alone administration, and serine and propanoic acid were unique biomarkers for the toxicities of CPF plus Cd administrations. Our results suggest that subchronic exposure to CPF and Cd alone, or in combination at their low doses, could cause disturbance of energy and amino acid metabolism. Overall, we have shown that analysis of serum metabolomics can make exceptional contributions to the understanding of the toxic effects following long-term low-dose exposure of the organophosphorus pesticide and heavy metal.

## Introduction

Chlorpyrifos (CPF) is a widely used organophosphorus insecticide with cholinergic toxicity^1,2^. CPF exposure can induce severe toxicity in multiple organs^3^. Cadmium (Cd) is a persistent toxic metal with very long half-life^4^. Chronic Cd exposure induces toxicity in kidneys, liver, and bones^5–8^. Due to their toxicity to different target organs, CPF and Cd pose significant threats to public health.

CPF and Cd are widespread environmental toxicants which often coexist in water, air, and soil^3,9^. Given that environmental toxicants are widespread, organisms are seldom exposed to only one chemical. Thus, it is important to study the combined toxicity of multiple toxicants. More and more studies suggest that combined exposure to different chemicals can potentiate the toxicity of single chemical alone^2,10,11^. As the technology advances, new methodologies such as omics approaches are developed, and great progress has been made in the study of mixture toxicology, which increases our ability to predict the potential risks of exposure to mixtures^12^.

The acute toxicity of CPF and Cd are well understood^13,14^. We have reported that chronic exposure to CPF and Cd individually and in combination caused neurotoxicity and hepatotoxicity^15,16^. We found that CPF and Cd disturbed energy and amino acids metabolism in the brain and liver. Therefore, in this study, we try to identify the biomarkers in serum, upon exposure to CPF, Cd, and their mixture.

Metabolomics is a very useful approach for toxicological studies because it can characterize the changes of metabolite levels induced by toxic chemicals and identify the specific biomarkers for each toxicant^17,18^. In the present study, we aim to use metabolomics analysis in serum to study the combined toxicity of CPF and Cd and identify potential biomarkers for their exposure. We use the gas chromatography - mass spectrometer (GC/MS) method, which has high sensitivity and specificity for detecting and quantification of chemicals in biofluids and tissue extracts^19^.

## Results

### Clinical serum biochemistry

In the high-dose Cd treatment group, the levels of serum biochemical parameters, including ALT, AST, TBIL, BUN, and CRE, significantly increased about 26%, 36%, 280%, 20%, and 34%, respectively, compared with the corresponding controls (Table 1). In the middle-dose Cd group, AST, TBIL, and BUN levels increased 41%, 368%, and 23%, respectively. However, only high dose CPF induced TBIL level (increased 560%) and middle-dose CPF induced AST level (increased 40%). In the low-dose individual CPF- or Cd-treated rats, there was no significant change of the parameters. For the combination treatments, AST and TBIL levels increased in most of the rats treated with CPF plus Cd, but ALT levels increased only in the rats exposed to the high-dose mixture of CPF and Cd. Intriguingly, the CPF plus Cd treatments did not alter BUN and CRE levels even though high dose of Cd alone induced significant increase in the BUN and CRE levels (Table 1).

**Table 1:** Changes of blood biochemical parameters in rats.

Groups	Parameters				
	ALT (U/L)	AST (U/L)	TBIL (µM)	BUN (mM)	CRE (µM)
Control	33.00 ± 0.77^bc^	112.33 ± 6.77^b^	0.25 ± 0.19^d^	5.27 ± 0.07 ^cd^	24.33 ± 1.29^b^
CPF-L	32.75 ± 1.80^bc^	142.75 ± 2.46^ab^	0.10 ± 0.04^d^	5.67 ± 0.05^abcd^	25.75 ± 0.67^b^
CPF-M	34.00 ± 2.71^bc^	157.67 ± 11.66^a^	0.47 ± 0.09 ^cd^	5.34 ± 0.23^bcd^	27.40 ± 0.87^ab^
CPF-H	36.67 ± 1.86^abc^	142.75 ± 10.23^ab^	1.65 ± 0.21^a^	5.01 ± 0.08 ^cd^	26.00 ± 1.93^b^
Cd-L	34.75 ± 1.23^bc^	148.25 ± 6.68^ab^	0.33 ± 0.14^d^	5.57 ± 0.26^abcd^	23.50 ± 0.58^b^
Cd-M	34.00 ± 1.18^bc^	158.00 ± 9.71^a^	1.17 ± 0.23^abc^	6.47 ± 0.37^a^	26.00 ± 0.77^b^
Cd-H	41.50 ± 3.38^a^	152.67 ± 8.27^a^	0.95 ± 0.24^abc^	6.30 ± 0.31^ab^	32.50 ± 2.08^a^
CPF-L + Cd-L	34.25 ± 0.43^bc^	170.50 ± 14.12^a^	0.90 ± 0.25^abc^	5.01 ± 0.15 ^cd^	27.40 ± 1.94^ab^
CPF-M + Cd-L	31.80 ± 0.80^bc^	164.50 ± 8.05^a^	0.77 ± 0.14 ^cd^	5.38 ± 0.18^bcd^	26.60 ± 1.50^ab^
CPF-H + Cd-L	32.75 ± 3.27^bc^	166.00 ± 15.38^a^	0.93 ± 0.24^abc^	5.94 ± 0.27^abc^	26.00 ± 0.97^b^
CPF-L + Cd-M	31.25 ± 0.99^c^	128.50 ± 6.71^b^	1.77 ± 0.41^ab^	5.73 ± 0.06^abcd^	27.50 ± 1.88^ab^
CPF-M + Cd-M	37.25 ± 2.64^abc^	149.75 ± 16.80^ab^	1.35 ± 0.24^ab^	5.02 ± 0.16 ^cd^	25.25 ± 1.88^b^
CPF-H + Cd-M	36.33 ± 1.69^abc^	168.67 ± 8.12^a^	0.23 ± 0.07^d^	5.42 ± 0.40^bcd^	28.00 ± 2.99^ab^
CPF-L + Cd-H	39.20 ± 2.11^ab^	153.20 ± 14.92^ab^	1.30 ± 0.39^abc^	5.14 ± 0.33 ^cd^	28.20 ± 0.80^ab^
CPF-M + Cd-H	37.75 ± 1.95^ab^	167.00 ± 14.59^a^	1.43 ± 0.25^abc^	4.82 ± 0.53^d^	27.25 ± 2.32^ab^
CPF-H + Cd-H	41.00 ± 2.10^a^	218.33 ± 27.36^a^	0.97 ± 0.07^bc^	4.79 ± 0.29^d^	26.80 ± 1.83^ab^

Note: Data were expressed as mean ± SE and differences among the groups evaluated by ANOVA. Different superscripted letters indicate a significant difference among the groups (P < 0.05), while the same letters indicate no significant difference among the groups (P > 0.05). Abbreviations: ALT, alanine aminotransferase; AST, aspartate aminotransferase; BUN, blood urea nitrogen; CRE, creatinine; TBIL, total bilirubin; BW, body weight; L, low dose; M, middle dose; H, high dose.

### The altered metabolic profiles in serum

**Figure 1:**
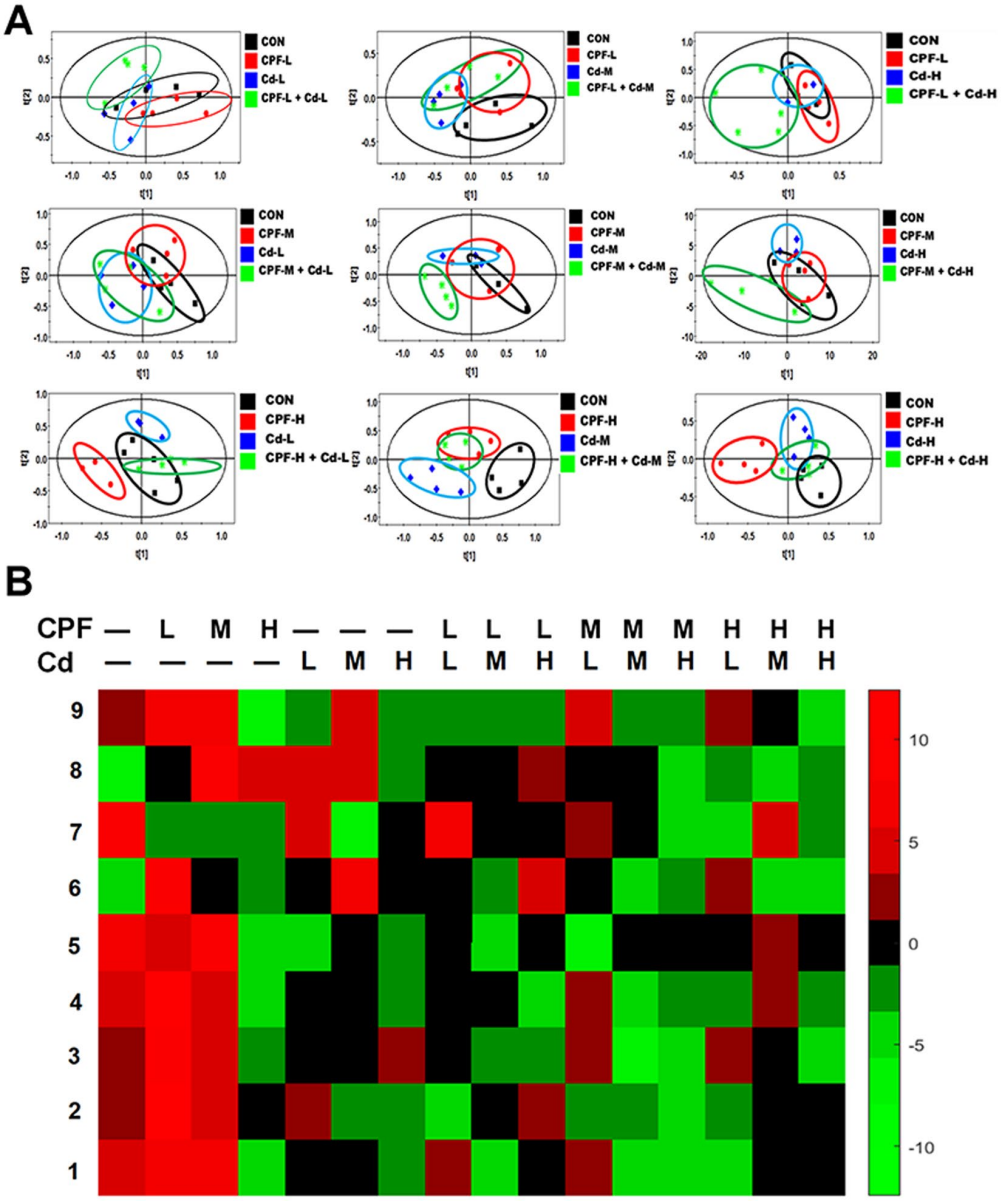
Metabolomic analysis of serum metabolites from rats. Rats were treated orally with chlorpyrifos (CPF) and cadmium (Cd) at doses of 1.7 and 0.7 mg/kg BW/day (low dose, L), 5 and 2 mg/kg BW/day (middle dose, M), and 15 and 6 mg/kg BW/day (high dose, H) and their combinations for 90 days. After the 90-day experimental treatment, blood samples were collected and the serum metabolites were determined by GC-MS. (**A**) The PLS-DA score plots of the serum metabolite profiles. Blank ellipse: control group; red ellipse: CPF-treated groups; blue ellipse: Cd-treated groups; green ellipse: CPF plus Cd-treated groups. t[1] indicated the first principal component; t[2] indicated the second principal component. (**B**) The hierarchically clustered heat map of the levels of 9 serum metabolites which changed significantly in the treated rats. The abbreviated letters and symbols in the upper and lower lines just above the heat map represent the CPF and Cd treatments respectively; “–” represents without treatment; L, M, and H represent the treatment with the corresponding chemicals, respectively, at low, middle, and high doses. The numbers on the left of the map represent the changed metabolites: 1: Propanoic acid; 2: Serine; 3: Aminomalonic acid; 4: Cysteine; 5: Glutamine; 6: Acetyl glutamine; 7: Fructose; 8: Gluconic acid; 9: Myo-inositol.

Then we carried out the metabolomics analysis with the serum samples from the rats. We first used PLS-DA analysis to explore the metabolic differences between treatment groups and the control group (Fig. 1A). Total 9 differential metabolites with VIP > 1 and significant differences among all groups (P < 0.05) were identified (Table 2). The heat map showed the change of these metabolites (Fig. 1B). As reported in a previous study, the data in the heat map was calculated by hierarchical clustering based on mean-centered and variance-scaled values [z-scores = (observed value – baseline median)/baseline standard variation]^20^. In the heat map, red (z-scores > 0) and green (z-scores < 0) indicated increased or decreased relative concentrations of the metabolites, respectively. The heat map showed that the trends of the change of serum metabolites after CPF or Cd individual treatment were opposite to the one after their combined treatment (Fig. 1B). The metabolomic analysis indicated that CPF and Cd mainly disturbed the amino acid metabolism pathways. CPF, Cd, and CPF plus Cd treatments decreased the concentrations of the most of the metabolites in serum compared with control.

**Table 2:** The AUC values of the serum metabolites that changed significantly in the treated rats compared with the control.

No.	Retention time (min)	Metabolites	P-value ^a^	AUC ^b^		
				CPF	Cd	CPF + Cd
1	9.24	Propanoic acid	0.006	0.472	0.278	**0.190**
2	16.63	Serine	0.000	0.722	0.222	**0.167**
3	19.52	Aminomalonic acid	0.001	0.528	**0.167**	0.286
4	22.12	Cysteine	0.000	0.556	0.250	0.214
5	24.18	Glutamine	0.016	0.361	**0.139**	**0.167**
6	27.44	Acetylglutamine	0.000	**0.833**	**0.861**	0.619
7	32.00	Fructose	0.002	**0.139**	0.306	**0.167**
8	37.48	Gluconic acid	0.011	**0.917**	**0.861**	0.786
9	40.25	Myo-inositol	0.010	0.472	0.417	0.405

^a^P values (treatment vs control) were calculated using ANOVA.

^b^AUC values (treatment vs control) were calculated using ROC analysis. Metabolites with 0.8 < AUC ≤ 1 or 0 ≤ AUC < 0.2 could discriminate treatment groups with control group with high accuracy and thus were selected as biomarkers. Levels of biomarkers with AUC > 0.8 were increased in the treatment groups. And levels of biomarkers with AUC < 0.2 were decreased in the treatment groups compared with the control group. Abbreviations: AUC, area under curve; Cd, cadmium; CPF, chlorpyrifos; CPF + Cd, chlorpyrifos plus cadmium.

### Identification and validation of serum metabolite biomarkers

**Figure 2:**
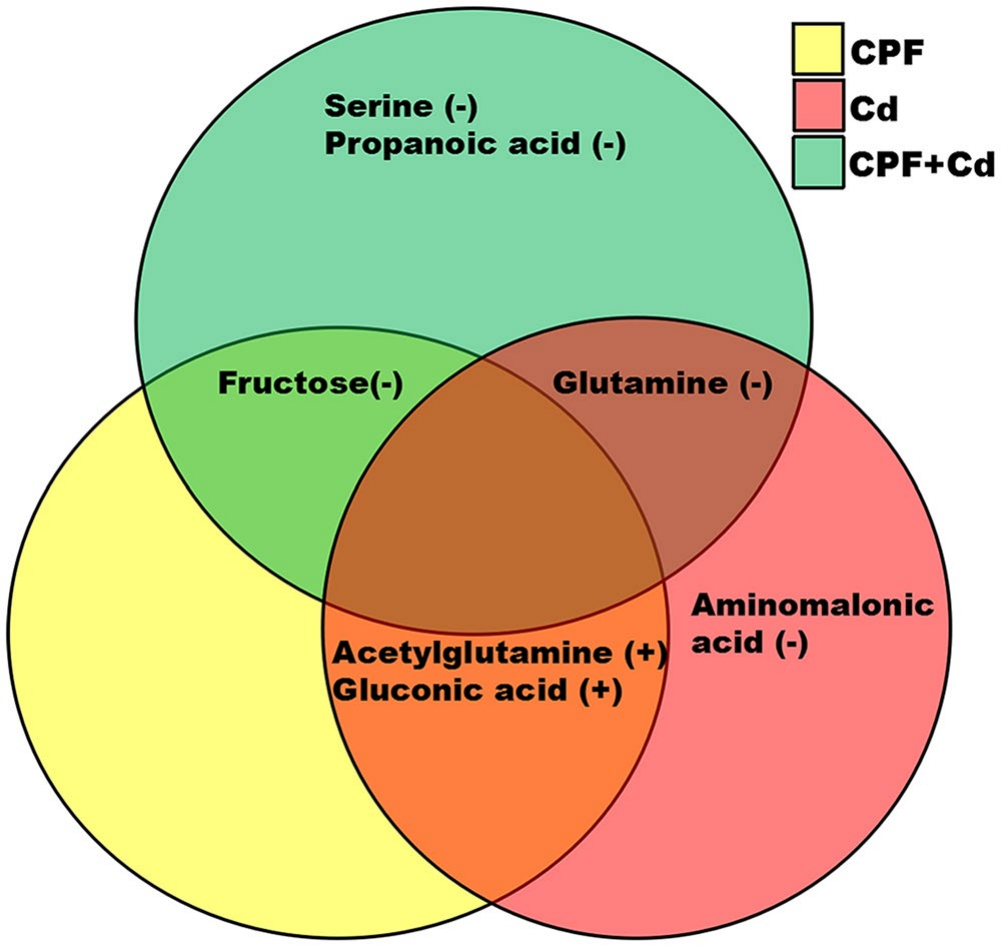
Identification of serum metabolite biomarkers. After the 90-day experimental treatment, blood samples of the exposed rats were collected and the serum metabolites were determined by GC-MS. The summary diagram of serum biomarkers with AUC > 0.8 or AUC < 0.2 in rats treated with Cd (red), CPF (yellow), or both (green). The areas of red, green, and yellow circles with no overlap represent the unique biomarkers for treatments with Cd, CPF, and their combinations, respectively. The “+” and “−” in parentheses indicate that the biomarkers were increased or decreased in the treated rats, respectively. Abbreviations: Cd, cadmium; CPF, chlorpyrifos.

**Figure 3:**
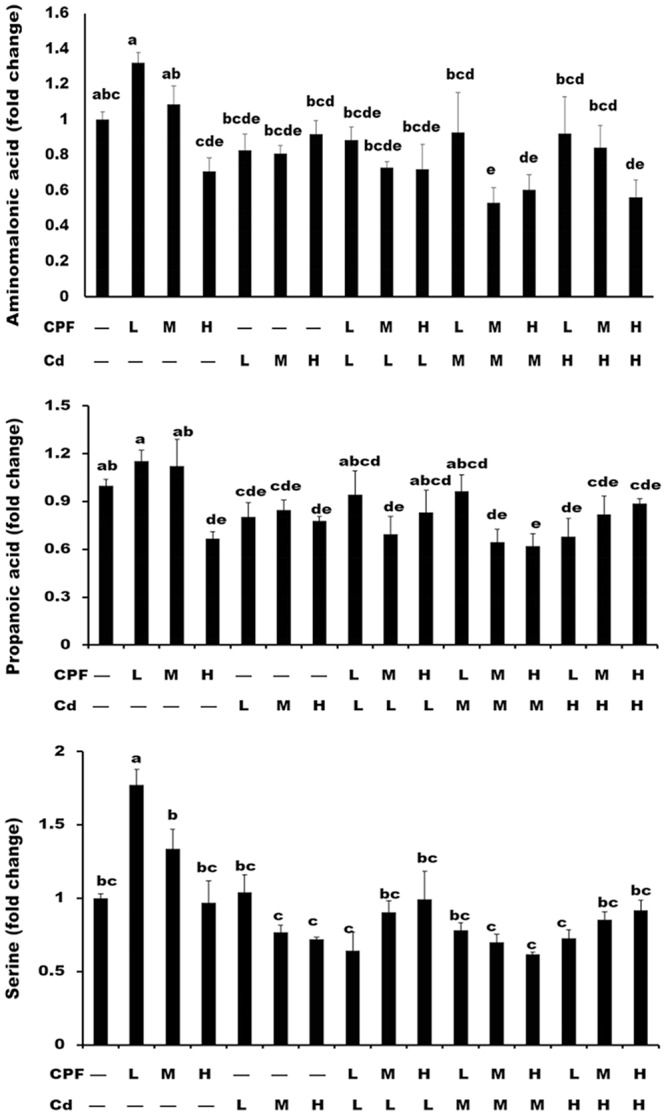
Levels of biomarkers in serum. The levels of the metabolites serine (**A**), propanoic acid (**B**), and aminomalonic acid (**C**) in serum of rats administrated orally with chlorpyrifos (CPF) and cadmium (Cd) at respective doses of 1.7 and 0.7 mg/kg BW/day (low dose, L), 5 and 2 mg/kg BW/day (middle dose, M), 15 and 6 mg/kg BW/day (high dose, H) and their combinations for 90 days. The abbreviated letters and symbols in the upper and lower lines just below the X-axis represent the CPF and Cd treatments respectively; “–” represents without treatment; L, M, and H represent the treatment with the corresponding chemicals, respectively, at low, middle, and high doses. Abbreviations: BW, body weight; Cd, cadmium; CPF, chlorpyrifos; L, low dose; M, middle dose; H, high dose.

To identify metabolite biomarkers for diagnosis of the toxicity of CPF, Cd, and CPF plus Cd, we performed a stepwise optimization algorithm based on receiver operating characteristic (ROC) as used in the previous studies^21,22^. Out of the total 9 altered metabolites, we identified 7 metabolites as potential biomarkers (serine, propanoic acid, fructose, glutamine, aminomalonic acid, acetylglutamine, and gluconic acid) (Table 3; Fig. 2) with “0.8 < AUC ≤ 1.0” or “0 ≤ AUC < 0.2”. These metabolites are mainly chemicals in the energy and amino acid metabolism (Fig. 3). Only one metabolite (decreased concentration of aminomalonic acid) was identified as the unique potential biomarker for Cd alone (Table 2; Figs. 2 and 3). Intriguingly, two metabolites, i.e., decreased concentrations of propanoic acid and serine (Fig. 3), were identified as unique biomarkers in the CPF plus Cd-treated rats (Table 3; Figs. 2 and 3).

**Table 3:** Serum biomarkers identified from rats treated with low dose of CPF, Cd alone and in combination.

Major metabolites	Retention time (min)	CPF	Cd	CPF + Cd
Propanoic acid	9.24	↑	↓ ^*^	—
Serine	16.63	↑^*^	—	↓
Aminomalonic acid	19.52	—	↓	↓^*^
Glutamine	24.18	—	↓^*^	↓^*^
Acetylglutamine	27.44	↑	↑	—
Fructose	32.00	↓^*^	—	↓^*^
Gluconic acid	37.48	↑^*^	↑^*^	↑^*^

Note: Comparisons among groups of rats were performed by ANOVA with post hoc analysis using Dunnett’s test. A *P* value < 0.05 was considered significant. Changes are relative to control samples: —, no change; ↓, decrease; ↑, increase; ^*^P < 0.05 compared with control. Abbreviations: Cd, cadmium; CPF, chlorpyrifos; CPF + Cd, chlorpyrifos plus cadmium.

### Cd affected the metabolism of CPF

**Figure 4:**
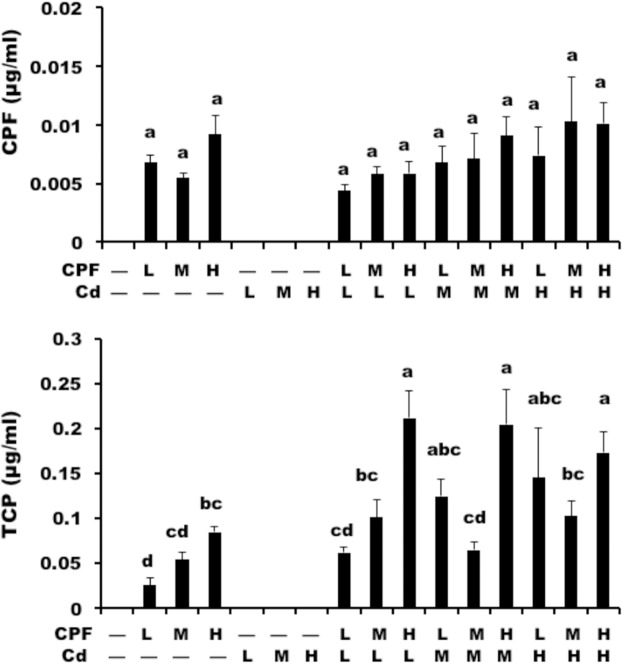
The levels of CPF and its metabolite TCP in serum of rats. The rats were administrated orally with CPF, Cd at doses of 1.7 and 0.7 mg/kg BW/day (low dose, L), 5 and 2 mg/kg BW/day (middle dose, M), 15 and 6 mg/kg BW/day (high dose, H), respectively, and their combinations for 90 days. After the 90-day administration, the blood samples were collected and the levels of CPF and its metabolite TCP in serum were measured by using HPLC method. Data were expressed as mean ± SE and the differences among different groups evaluated by ANOVA. Different superscripted letters (**a–d**) indicate a significant difference among groups (P < 0.05), while the same letters indicate no significant difference among groups (P > 0.05). Abbreviations: BW, body weight; L, low dose; M, middle dose; H, high dose; Cd, cadmium; CPF, chlorpyrifos; TCP, 3,4,5-trichloropyrindinol.

To investigate the mechanism of CPF and Cd’s interaction, we determined whether CPF and Cd affected the metabolism of each other. Unfortunately, Cd in the serum was not detected even in the samples from the rats treated with high-dose Cd, probably due to the low dose of Cd administration. Nevertheless, we could detect CPF and TCP, the final product of CPF in the serum samples. We found that compared with the rats treated with CPF alone, the concentration of CPF decreased slightly in the CPF plus Cd-treated groups (Fig. 4, upper). And the levels of TCP increased significantly after CPF plus Cd treatment, compared with the CPF alone treatment (Fig. 4, lower). Thus, our results indicated that Cd affected the metabolism of CPF.

## Discussion

Nowadays, metals and organophosphorus pesticides are commonly found to co-exist in the environment^23,24^. Thus, more research is needed to reveal the combined toxic effect of CPF and Cd. Interestingly, CPF and Cd have very different modes of action in the organisms. CPF inhibits the activity of acetylcholinesterase^25,26^. Cd causes the nephrotoxicity in the body^27,28^. In this study, we used GC-MS-based metabolomics approach to analyze the change of metabolites, and ICP-MS and HPLC methods to determine the levels of Cd, CPF and its metabolic product TCP in serum to study the mechanism underlying the combined toxicity of CPF and Cd. The heptadecanoic acid was used as the internal standard of GC-MS analysis to ensure the accuracy of metabolites analysis. For ICP-MS and HPLC analysis, we also used the standards of Cd, CPF and TCP to make the calibration curves. The R^2^ values of the calibration curves are all greater than 0.999. Additive responses, in which the effect of combinations of chemicals were neither stronger nor weaker than the sum of the effect of single chemical response, would be expected^22^. However, our results indicate that CPF plus Cd likely will not have stronger effect on the disturbance of metabolism than the sum effect of individual chemicals. The changed blood biochemical parameters indicate the liver and kidney damage after CPF or Cd treatment. The changes of levels of these parameters in the CPF plus Cd groups were less than the sum of those in CPF or Cd groups, especially for ALT, TBIL, and BUN (Table 2). The change of biomarkers showed that the toxic response of the combinations of CPF and Cd was less than additive effect (Figs. 1B and 3), because more metabolites in CPF plus Cd treatments in the heat map (Fig. 1B) had black or light red/green color compared with those in the CPF alone or Cd alone treatments. Thus, these results indicate that there is mainly an antagonistic interaction between CPF and Cd because when the response is less than the sum of the individual effects, their interaction is characterized as antagonistic^29^. By determining the serum levels of Cd and CPF, we found that compared with the rats treated with CPF alone, the concentration of CPF decreased slightly in the CPF plus Cd-treated groups (Fig. 4). And the levels of TCP, a metabolic product of CPF, increased significantly after CPF plus Cd treatment, compared with the CPF alone treatment (Fig. 4). Thus, the interference of CPF metabolism by Cd may lead to the antagonistic interaction between CPF and Cd.

By using GC-MS-based metabolomics method, we demonstrated for the first time that CPF and Cd significantly altered the metabolic profile in the rat serum. Nine serum metabolites were significantly changed after treatments. In the serum samples from the CPF-, Cd-, and CPF plus Cd-treated animals, we detected the evident alteration in a series of metabolites, which were mainly derived from the amino acids metabolism (glutamate, serine, and cysteine metabolism). There were also a few changes in energy metabolism and tricarboxylic acid cycle (TCA cycle) (fructose, gluconic acid, and propanoic acid).

Propanoic acid is an important glycolysis product, and it can be metabolized into propionyl-CoA and then enters the TCA cycle^30^. Thus, the decrease of propanoic acid indicates disruption in energy metabolism by CPF and Cd treatment. Aminomalonic acid has important biological functions to impart calcium binding activity to proteins^31^. In addition, it is reported that aminomalonic acid can inhibit asparagine synthesis^32^. Several metabolomics studies have also found that altered aminomalonic acid level in serum was associated with neuropsychiatric disorders, ketamine overdose and aneurysm, indicating that aminomalonic acid is an important serum indicator for diseases and toxicities^30,33,34^. Serine is an important amino acid and the precursor for choline synthesis^35^. Actually, in our previous study, we found that serine was also a unique biomarker for CPF plus Cd in brain of rats^2^. Compared with the changes of metabolites in liver^16^, CPF, Cd, and CPF plus Cd induced the alteration of the similar metabolic pathways (alanine, aspartate, and glutamate metabolism; glycine, serine, and threonine metabolism; phenylalanine, tyrosine, and tryptophan biosynthesis; phenylalanine metabolism) in the serum.

As far as we know, no serum biomarkers are available to diagnose the toxicosis of CPF, Cd, and CPF plus Cd at their low doses. The observation of body weight and relative organ weight in all treatment groups suggests that CPF and Cd cause unobvious poisoning in the treated groups, except at high dose of CPF^15,16^. For the blood biochemical analysis, overall, no obvious change was observed in the low-dose individual CPF- or Cd-treated groups although high-dose Cd induced the increase of the tested parameters and the middle dose of Cd, CPF, or Cd plus CPF caused changes of only some of the parameters. However, serum metabolomics analysis revealed that some metabolites significantly changed even at low dose of CPF, Cd, and their combinations before the toxic signs and pathological tissue damage could be observed. Given the observation of toxic signs, regular blood biochemical analysis, and metabolomics analysis, the changes of serum metabolites were more sensitive to the toxicity of the tested chemicals. Although in our study, the fold change of biomarkers was relatively low in the serum, a previous study showed that even small change of the metabolites may indicate severe damage^36^.

In conclusion, with the use of GC-MS analysis-based method, we identified the serum metabolite biomarkers for CPF, Cd, and their combinations with high accuracy and further identified metabolic differences between treatment groups and controls. In the serum, aminomalonic acid is the biomarker for the Cd alone-treated and serine and propanoic acid are unique biomarkers for the CPF plus Cd-treated animals.

## Materials and Methods

### Chemicals

Hexane and methanol were chromatographic grade and obtained from Dikma Technologies Inc. (Beijing, China). Barbitalum natricum was purchased from Beijing Chemical Plant (Beijing, China). Thiobarbituric acid, 3,4,5-trichloropyrindinol (TCP), guanidine hydrochloride, methoxyamine, methyl-trimethyl-silyl-trifluoroacetamide (MSTFA) and trimethylchlorosilane (TMCS) were obtained from J & K Chemical Ltd (Beijing, China). Chlorpyrifos (purity > 96%) was obtained from Nantong Shuangma Fine Chemical Co., Ltd (Jiangsu, China). Cadmium chloride was obtained from Sigma-Aldrich Chemical Company (St Louis, MO, USA). All other reagents were obtained from commercial providers.

### Animals and treatment

The animal experiment was carried out in the same way as our early reports^15,16^. Eighty male Sprague-Dawley (SD) rats (6–8 weeks old) were obtained from Beijing HFK Bioscience Co., Ltd (Beijing, China) and were housed under a specific-pathogen-free (SPF) condition with 22 ± 2 °C of the animal room, 50%-60% humidity and a light/dark cycle of 12 h. Animals had free access to water and the diet which is a commercially prepared laboratory animal diet (HFK Bioscience, Beijing, China).

The rats were randomly grouped and treated with chlorpyrifos (CPF), cadmium (Cd) and their combinations in the same way as reported in our earlier study^15^. Previous studies showed the acute oral half-lethal doses (LD_50_) of chlorpyrifos and cadmium were 229 mg/kg body weight (BW) and 88 mg/kg BW for rats, respectively^2,7^. So in this study we chose the doses of 1/135 LD_50_, 1/45 LD_50_, and 1/15 LD_50_ of each chemical as low-, middle-, and high- dose for the treatment groups, respectively. The doses (mg/kg BW/day) used were: CPF 1.7 (low dose), 5 (middle dose), and 15 (high dose); Cd 0.7 (low dose), 2 (middle dose), and 6 (high dose). Experimental design for the combined effects of CPF and Cd was summarized in Supplementary Table S1. Each group contained five animals. The low doses of CPF and Cd used in this study (1.7 and 0.7 mg/kg BW/day, respectively) were chosen based on human occupational and environmental exposure to CPF and Cd. The calculation was described in our previous study^15^.

The chemicals were given to the animals in the same way as the early reports^15,16^. Chlorpyrifos and cadmium chloride were dissolved in corn oil and deionized water, respectively, and administered via oral gavage (0.5 mL/kg BW/day). The rats in Cd groups were also given equivalent volume of corn oil, and the rats in CPF groups were also given equivalent volume of deionized water. Rats were given these chemicals for 90 days. The rats received an equivalent volume of corn oil and water served as control. All animal procedures were carried out in accordance with China legislation and approved by the Animal and Medical Ethics Committee from Institute of Zoology of Chinese Academy of Sciences.

### Sample preparation

The sample preparation was carried out in the similar way as our early report^37^. Briefly, twenty-four hours following the final dose of 90-day administration, rats were subjected to anesthesia with barbitalum natricum. Then, 5 mL of blood samples were collected from all animals. For each blood sample, serum samples were separated by centrifugation and then divided into two aliquots (about 1 mL each). One aliquot was used for biochemistry test and the other was stored at −80 °C for GC-MS analysis.

### Serum biochemical analysis

The clinical biochemical parameters including alanine aminotransferase (ALT), aspartate aminotransferase (AST), blood urea nitrogen (BUN), creatinine (CRE), and total bilirubin (TBIL) in serum were determined by the Autolab-PM4000 automatic analyzer (AMS Co., Rome, Italy) with the standard spectrophotometric methods. Values of the serum biochemical parameters were expressed as the mean ± SD.

### Sample preparation for GC-MS analysis

The serum was dissolved at 37 °C and swirled before use. Eight hundred microliters (800 µL) of methanol was added to a mixture of 100 µL of serum, 100 µL of water and 10 µL heptadecanoic acid (6 mg/mL), and then the mixture was swirled for 10 s, kept on ice for 10 min and centrifuged at 12,000 × g for 10 min at 4 °C. The supernatant was transferred, and dried in a vacuum concentrator.

The sample preparation procedure was the same as our earlier report^15^. Briefly, fifty microliters of methoxyamine in pyridine were added to the dried metabolites extract and vigorously vortexed. After incubated for 16 h at room temperature, the samples were trimethylsilylated for another 1 h by adding 100 µL of MSTFA with 1% TMCS^38,39^. Finally, 200 µl hexane was added, and the samples were vigorously vortexed again, centrifuged at 12,000 × g for 15 min and the supernatant was transferred to the GC sample vials for GC/MS analysis. Heptadecanoic acid was used as internal standards.

### GC-MS analysis

The GC-MS analysis was carried out as reported in our previous study^15^. Gas chromatography was performed on a 6890 N gas chromatograph system (Agilent Co., Palo Alto, CA, USA) equipped with a HP-5 MS capillary column (60 m × 0.25 mm × 0.25 µm)^39^. The injection temperature was 250 °C. The carrier gas flow rate was 1 mL/min. The column temperature was held at 90 °C for 1 min and then raised to 175 °C at the rate of 5 °C/min and held for 3 min. The temperature was subsequently raised to 270 °C at the rate of 3 °C/min and then to 310 °C at the rate of 20 °C/min. And then the temperature was maintained at 310 °C for 15 min. The temperatures of the transfer interface and the ion source were 250 and 200 °C, respectively. Ions were generated by a 70 eV electron source at the full scan mode (*m/z* 40–600), with the acquisition rate of 20 spectra/s.

### Metabolomics data analysis

The metabolomics data analysis was carried out in a similar way as reported in our previous study^15^. Different metabolites of rat serum were identified by NIST library 2005. And automatic mass spectral deconvolution & identification system (AMDIS) was used to address spectral convolution, and peaks with signal-to-noise ratio higher than 3 were selected for further analysis. Data pretreatment procedures for metabolites were performed by Matlab 7.1 (The MathWorks, Inc., Natick, MA, USA) to normalize the peak area of each metabolite to that of the internal standard heptadecanoic acid. Partial least squares-discriminant analysis (PLS-DA) was used for multivariate statistical analysis with SIMCA-P 11.5 software (Umetrics, Umeå, Sweden). In order to select the candidate biomarkers, the variable importance in the project (VIP) values were calculated by using SIMCA-P software. The metabolites data were checked to have a normal distribution by using SPSS18.0 software (SPSS, Inc., Chicago, MI, USA). Thus, the significance among groups for these candidate biomarkers was calculated by using analysis of variance (ANOVA) with F-test in SPSS 18.0 software and only those with P < 0.05 were selected.

Finally, to determine the accuracy of the biomarkers to discriminate the treated groups with the control group, the area under the curve (AUC) value for receiver-operating characteristic (ROC) curve was calculated using SPSS 18.0 software. The detailed meanings of AUC values are: AUC = 0.5: not accurate, 0.5 <AUC ≤ 0.7 or 0.3 ≤ AUC < 0.5: less accurate, 0.7 < AUC ≤ 0.9 or 0.1 ≤ AUC < 0.3: moderately accurate, 0.9 <AUC < 1.0 or 0 < AUC < 0.1: highly accurate; AUC = 1 or 0: super accurate^21^. Thus, considering accuracy and the actual distribution of the data, we chose the metabolites with 0.8 < AUC ≤ 1.0 or 0 ≤ AUC < 0.2 as potential biomarkers in this study.

### Measurement of cadmium level in serum

The levels of Cd in the serum of rats were determined by the inductively coupled plasma mass spectrometry (ICP-MS) according to the method reported in a previous study^40^. Briefly, the serum samples were digested with a mixture of nitric acid and hydrogen peroxide (3/2, v/v) for 20 min at 180 °C by microwave. Then the concentrations of Cd were quantified by Agilent 7500 ICP-MS (USA).

### Measurement of chlorpyrifos and its metabolite levels in serum

The assay was carried out by the method reported by the previous study^41^. Briefly, the serum samples were mixed with methanol and then centrifuged. The supernatants were collected, dried under nitrogen and then dissolved in 150 μL methanol. The samples were analyzed by the Agilent 1100 series HPLC system (Santa Clara, CA, USA) with UV detector. Calibration standards of CPF and TCP (3,4,5-trichloropyrindinol) were prepared in acetonitrile with concentrations ranging from 0.1 to 10 mg/mL. Linear calibration curves were obtained by plotting the peak areas of CPF or TCP as a function of the concentration using the Excel program (Microsoft Corp., Redmond, WA). LOD for CPF was 0.05 mg/mL and LOQ for CPF was 0.1 mg/mL. LOD and LOQ for TCP were 0.05 mg/mL was 0.1 mg/mL respectively.

### Statistical analysis

SPSS 18.0 software (SPSS, Inc., Chicago, MI, USA) was employed for the statistical evaluation. The raw data were checked to have a normal distribution by using Kolmogorov-Smirnov test in SPSS. Analysis of variance (ANOVA) was performed to access significant difference among the groups, followed by post hoc Dunnett’s test. The difference was considered significant with the value of P < 0.05.

## Supplementary information

Supplementary Information.

## Data Availability

All data generated or analyzed during this study are included in this published article and its Supplementary Information file.
