# Supplementary material for: Identification of metabolite biomarkers in serum of rats exposed to chlorpyrifos and cadmium
Source: Sci Rep. 2020 Mar 19;10:4999. doi: 10.1038/s41598-020-61982-4 (PMC7081290; doi:10.1038/s41598-020-61982-4)
Supplement: Supplementary file 1 — Supplementary Information. [file 41598_2020_61982_MOESM1_ESM.pdf]

Identification of metabolite biomarkers in serum of rats exposed to chlorpyrifos and cadmium

Ming-Yuan Xu, Pan Wang, Ying-Jian Sun, Lin Yang, Yi-Jun Wu

Supplementary Table S1. Experimental design for combined effects of chlorpyrifos and cadmium

Groups	Chlorpyrifos (mg/kg BW/day)	Cadmium (mg/kg BW/day)
Control	—	—
CPF-L	1.7	—
CPF-M	5	—
CPF-H	15	—
Cd-L	—	0.7
Cd-M	—	2
Cd-H	—	6
CPF-L + Cd-L	1.7	0.7
CPF-M + Cd-L	5	0.7
CPF-H + Cd-L	15	0.7
CPF-L + Cd-M	1.7	2
CPF-M + Cd-M	5	2
CPF-H + Cd-M	15	2
CPF-L + Cd-H	1.7	6
CPF-M + Cd-H	5	6
CPF-H + Cd-H	15	6

Note: Sprague-Dawley rats were administrated orally with chlorpyrifos (CPF), cadmium (Cd) at doses of 1.7 and 0.7 mg/kg BW/day (L), 5 and 2 mg/kg BW/day (M), 15 and 6 mg/kg BW/day (H), respectively, and their mixtures for 90 days. CPF and Cd were dissolved in corn oil and deionized water, respectively, and administered via oral gavage (0.5 ml/kg BW). The rats received an equivalent volume of corn oil and water served as control. Abbreviations: BW, body weight; L: low level; M: middle level; H: high level.
